# Translation and cultural adaptation of the stroke impact scale 2.0 (SIS): a quality-of-life scale for stroke

**DOI:** 10.1590/1516-3180.2017.0114281017

**Published:** 2018-01-09

**Authors:** Aline Dias Brandão, Natasha Bertocco Teixeira, Maria Claudia Brandão, Milena Carlos Vidotto, José Roberto Jardim, Mariana Rodrigues Gazzotti

**Affiliations:** I MSc, PhD. Research Fellow, Respiratory Division and Pulmonary Rehabilitation Center, Hospital São Paulo, Escola Paulista de Medicina (EPM), Universidade Federal de São Paulo (Unifesp), São Paulo (SP), Brazil.; II PT. Physiotherapist and Former Research Fellow, Respiratory Division and Pulmonary Rehabilitation Center, Hospital São Paulo, Escola Paulista de Medicina (EPM), Universidade Federal de São Paulo (Unifesp), São Paulo (SP), Brazil.; III PT. Physiotherapist and Former Research Fellow, Respiratory Division and Pulmonary Rehabilitation Center, Hospital São Paulo, Escola Paulista de Medicina (EPM), Universidade Federal de São Paulo (Unifesp), São Paulo (SP), Brazil.; IV MD, PhD. Associate Professor, Physiotherapy Department, Universidade Federal de São Paulo (Unifesp), São Paulo (SP), Brazil; V MD, PhD. Associate Professor, Respiratory Division and Pulmonary Rehabilitation Center, Hospital São Paulo, Escola Paulista de Medicina (EPM), Universidade Federal de São Paulo (Unifesp), São Paulo (SP), Brazil.; VI MSc, PhD. Research Fellow, Respiratory Division and Pulmonary Rehabilitation Center, Hospital São Paulo, Escola Paulista de Medicina (EPM), Universidade Federal de São Paulo (Unifesp), Brazil.

**Keywords:** Validation studies, Quality of life, Translations, Stroke

## Abstract

**BACKGROUND:**

No specific quality-of-life scale for stroke patients has previously been translated and evaluated for reproducibility, for use in the Portuguese language. Internationally, the instrument for this purpose is the Stroke Impact Scale 2.0 (SIS). Use of of SIS enables comprehensive analysis on the impact of mild and moderate stroke on patients’ lives. The aims here were to translate SIS into Portuguese, adapt it culturally, evaluate its reproducibility and correlate it with SF-36 among stroke patients.

**DESIGN AND SETTING:**

Translation and validation study.

**METHODS:**

The process of initial and retrograde translation was performed, in addition to cultural adaptation to the Brazilian language and culture. SIS was applied to 40 patients, who answered the questions three times. On the first day, the scale was applied twice by two independent researchers (to evaluate interobserver reproducibility). Fifteen days later, the scale was applied for a third time by another researcher (intraobserver reproducibility). The intraclass correlation coefficient (ICC) was used to measure the reproducibility of the SIS scale.

**RESULTS:**

The reproducibility of the whole scale was very good (ICC: 0.73 to 0.99). Intraobserver reproducibility in all domains was also very good (ICC: 0.85 to 0.95). Comparison of SIS with SF-36 showed that the domains of strength, mobility and activities of daily living (ADLs) correlated moderately with the functional capacity domain, as did the ADL domain with general health status. The other correlations were weak. The depression domain showed a moderate negative correlation with the memory and communication domains.

**CONCLUSION:**

The translation of the SIS 2.0 scale was easy to understand and it had good reproducibility among stroke patients.

## INTRODUCTION

Among chronic non-communicable diseases, those of the circulatory system are the main cause of mortality worldwide, including Brazil, which has one of the highest rates in South America. Among cardiovascular diseases (CVDs), cerebrovascular disease has specific characteristics within Brazilian realities and is one of the most neglected diseases in the country.^1^

The incidence of stroke is increasing due to increased life expectancy and changes in lifestyle. It has been estimated that in South America it will become more evident over the next decades for the same reasons. Stroke mortality in Brazil has been reported to be the highest in South America for both sexes.^2^

Stroke has major impacts on individuals’ sensory motor function and gives rise to disorders of language comprehension and orientation. When death does not occur, stroke has multiple negative consequences on individuals’ lives, such as institutionalization, great dependence on other people and cognitive and communicative impairment.^3^ It gives rise to a great need for care, since it affects human functions, and it disrupts not only the patient’s life but also the lives of the entire family because of its sequelae. Most stroke survivors are left with permanent sequelae for which constant care is required. However, the implications of these consequences on the quality of life of these patients have not yet been assessed in depth, and neither have the prospects for these patients. Evaluations on stroke have usually been limited to neurological impairment and disability. Measuring the quality of life (QOL) after stroke could provide a spectrum of related answers for the many issues surrounding stroke.^4^

According to the World Health Organization (WHO), the concept of health is not merely the absence of disease, but the individual’s perception of complete physical, mental and social wellbeing.^5^ Health-related QOL is investigated through self-evaluation by patients across multiple dimensions that are not limited to physical, social and emotional concepts. Measuring QOL is potentially more relevant to patients than are measurements of impairment or disability. QOL is also an important prognostic indicator for stroke, and it enables a broader description of the disease.^6^

Evaluating health and the effects of treatments involves assessing changes to the frequency and severity of diseases and estimating wellbeing. One way to assess patients’ wellbeing is through quality-of-life questionnaires. Instruments for measuring quality of life are a useful way to transform subjective measurements into objective data that can be quantified and analyzed, and are important for checking the impact of interventions on patients’ health and quality of life.^7^

There are few specific quality-of-life questionnaires for patients with stroke. The scales that have most commonly been used to measure these results are the Rankin scale, Barthel index and National Institutes of Health stroke scale (NIHSS).^8^^,^^9^^,^^10^ However, these are not sensitive enough to assess mild stroke and do not assess quality of life dimensions such as mood, communication and function. Moreover, no quality-of-life questionnaire specific for stroke patients has been translated and validated for use in the Portuguese language. Internationally, the instrument for this purpose is the Stroke Impact Scale 2.0 (SIS).^11^

The aim of this study was to translate the SIS questionnaire into Brazilian Portuguese, to perform cultural adaptation on it and to evaluate its reproducibility.

## METHODS

### Patients

The study included 40 clinically stable patients who had been diagnosed with stroke at Escola Paulista de Medicina (EPM) - Universidade Federal de São Paulo (Unifesp), according to their consecutive arrival at the outpatient clinic.

The patients selected were over 18 years of age and had a minimum score in the Mini-Mental State Examination (MMSE) ≥ 24 for literate individuals and ≥ 13 for illiterate individuals. Patients who were admitted to the hospital, experienced changes in medication and/or did not participate in all evaluations were excluded.

The patients signed a free and informed consent statement and the protocol was approved by the Ethics Committee for Medical Research of Hospital São Paulo - Unifesp (no. 582/08).

#### Questionnaire structure

The SIS consists of 64 questions divided into eight areas and an independent measurement of the patient’s overall perception of his or her percentage recovery after stroke, graded from zero (no recovery) to 100 (full recovery), in a format similar to a visual analogue scale. The domains are: strength, hand function, mobility, activities of daily living, instrumental activities of daily living, memory, communication, mood and social participation. The number of questions in each domain ranges from 4 to 11 and their scores range from 5 to 1, according to the degree of difficulty, amount of time and strength expended, depending on the dimension. The higher the score is, the better the quality of life is. Four of these dimensions (hand function, mobility, activities of daily living and instrumental activities of daily living) can be evaluated together to form a single domain called the physical domain. The SIS uses the scoring algorithm from the Medical Outcomes Study 36-item Short-Form Survey (SF-36) and is scored as follows for each dimension:

$$Scoring:\;\frac{\left[\left(real\;score-lowest\;possible\;score\right)\right]\times 100}{possible\;score\;amplitude}$$

This scale is aimed towards evaluating other important issues, i.e. other than physical issues, that can substantially interfere in the quality of life of stroke patients from the patients’ own point of view or that of their caregivers.

### Translation into Portuguese and cultural adaptation

In the first stage of this study, two local translators who are native speakers of the Portuguese language and bilingual in English were recruited. Two versions of the questionnaire in Portuguese were produced by them.

In the second stage, a third person, who was also a native Portuguese speaker and bilingual in English, carried out an evaluation on the two translations and reconciled them into a single version.

In the third stage, a fourth translator, who did not have access to the original version of the questionnaire, translated the reconciled version back to the original language (English). So far, the translation process was similar to that used for the translation of the Saint George Questionnaire for Respiratory Diseases,^12^ Airways Questionnaire (AQ 20)^13^ and functional assessment of cancer therapy-brain (FACT-BR).^14^

In the fourth stage, the group responsible for the research compared the version translated into English with the original version in order to detect any friction relating to culture, thus generating a Portuguese version that would be applied to a sample of patients.

This questionnaire and an interview were applied to 10 consecutive patients who were being followed up at the neurovascular clinic of EPM-Unifesp, and these patients participated in the stage of translation and cultural adaptation of the questionnaire.

The aim of the interview was to evaluate the difficulties that patients might have in understanding the questionnaire and verify patients’ interpretations in all areas. In the event of any problems, the interviewer would need to find an alternative means for testing or conversions, or ask the individual to suggest an alternative. After the questionnaire had been applied to these patients and final corrections had been made, the questionnaire was deemed to have reached its final version.

### Reproducibility assessment on SIS 2.0

To assess reproducibility, the SIS 2.0 questionnaire (final release) was applied to 40 patients from the previous stage. The questionnaire was administered three times to each patient. Only two researchers participated in administering the questionnaires. At the first appointment, SIS 2.0 was applied twice on the same day, at different times by two different researchers (researchers 1 and 2) who did not have access to the responses from each other’s applications. This procedure was used to analyze interobserver reproducibility by means of the agreement analysis, presented by the intraclass coefficient correlation (ICC). Cronbach’s alpha was also calculated. After a period of 15 days, SIS 2.0 was applied once again by researcher 1, to study intraobserver reproducibility.

### Statistical analysis

The data were analyzed regarding normality of distribution, using the Shapiro-Wilk test. The data showed parametric distribution and were expressed as means and 95% confidence intervals (95% CI). Descriptive statistical analysis was used for demographic and clinical characterization of the patients evaluated. To compare pairs of independent samples, we used the Student t test.

To measure reliability, we used the intraclass correlation coefficient (ICC) and presented the 95% confidence interval (95% CI). The ICC was characterized as follows: good reliability 0.80-1.0; fair reliability 0.60-0.79; and poor reliability < 0.60.^15^ The statistical significance level was set at P < 0.05. Statistical analyses were performed with the aid of the SPSS 17.0 software.

## RESULTS

A total of 52 stroke patients participated in this study: 52 were initially recruited, 2 were excluded (one was excluded because he did not return for the second visit, and one because he did not answer the questionnaire), thus resulting in a sample of 50 patients who completed the study. The sample only included two illiterate individuals, but the questionnaire had to be read out for all of the individuals assessed because their educational level was low and they had difficulty in reading. Out of the 50 patients who completed the study, 10 participated in the stage of translation and cultural adaptation of the questionnaire and 40 participated in the questionnaire reproducibility study. All of the patients remained clinically stable and the treatment was not changed during the interval of fifteen days between the questionnaire applications.

In the group that participated in the translation and cultural adaptation, five patients (50%) were female. The average age was 45.2 ± 11.4 years. For the reproducibility study, 23 patients (57.5%) were female. The patients’ characteristics are shown in Table 1.

**Table 1:** Demographic characteristics of the 40 patients evaluated for the assessment on reproducibility

Variables	Data
Age (years), mean ± SD	61.10 ± 11.5
Gender, n (%)	
Female	23 (57.5)
Male	17 (42.5)
MMSE score, mean ± SD	26.22 ± 3.33
Type of stroke, n (%)	
Ischemic	39 (97.5)
Hemorrhagic	1 (2.5)

SD = standard deviation; m = mean; n = absolute number; MMSE = mini-mental state examination.

The patients’ cognition was assessed by means of the MMSE questionnaire. The minimum score given was 20 and the maximum was 35. The average length of time taken to answer the MMSE questionnaire was 6.27 ± 2.26 minutes, with a range from 3 to 11 minutes.

The intraclass correlation coefficient for analysis of intraobserver variability (total scores assessed 15 days apart) showed the value of 0.95 (95% CI: 0.75-0.99). The analysis of interobserver variability assessed on the same day also showed ICC of 0.95 (95% CI: 0.73-0.99), and these two reproducibility values were considered to be very good (Table 2).

**Table 2:** Intraobserver reproducibility of each domain

	ICC	Confidence interval	P
Strength	0.86	(0.73-0.92)	< 0.0001
Hand function	0.99	(0.98-0.99)	< 0.0001
Mobility	0.92	(0.85-0.96)	< 0.0001
Activities of daily living	0.93	(0.86-0.96)	< 0.0001
Memory	0.89	(0.78-0.94)	< 0.0001
Communication	0.87	(0.75-0.93)	< 0.0001
Mood	0.75	(0.53-0.86)	< 0.0001
Social participation	0.93	(0.87-0.96)	< 0.0001

ICC = intraclass correlation coefficient; P = significance level.

The ICC from the analysis of reproducibility of the various domains assessed over a 15-day interval was very good and ranged from 0.75 to 0.99 (Table 2). The ICC from the analysis of interobserver reproducibility of the fields was also considered to be very good (0.73 to 0.99) (Table 3).

**Table 3:** Interobserver reproducibility of each domain

	ICC	Confidence interval	P
Strength	0.92	(0.85-0.96)	< 0.0001
Hand function	0.99	(0.98-0.99)	< 0.0001
Mobility	0.88	(0.77-0.94)	< 0.0001
Activities of daily living	0.96	(0.93-0.98)	< 0.0001
Memory	0.85	(0.71-0.92)	< 0.0001
Communication	0.84	(0.69-0.92)	< 0.0001
Mood	0.73	(0.50-0.86)	< 0.0001
Social participation	0.94	(0.89-0.97)	< 0.0001

ICC = intraclass correlation coefficient; P = significance level.

Also regarding the assessment of reproducibility, the averages for the eight domains were compared between the two times that the questionnaire was administered by the same researcher (Table 4) and on the same day by two different researchers (Table 5).

**Table 4:** Mean, standard deviation and difference between the two visits (V1 and V2)

	V1 mean ± SD	V2 mean ± SD	Δ	P
Strength	44.4 ± 3.8	45 ± 3.6	-0.625	0.81
Hand function	69.2 ± 5.2	69.4 ± 5.2	-0.144	0.89
Mobility	77.0 ± 3.3	81.0 ± 3.4	-4.063	0.03
Activities of daily living	81.4 ± 2.8	81.1 ± 3.2	-0.186	0.90
Memory	75.7 ± 2.9	80.3 ± 3.0	-4.523	0.02
Communication	81.6 ± 3.4	85.9 ± 2.7	-4.334	0.04
Mood	52.8 ± 2.3	58.7 ± 1.7	-5.833	0.01
Social participation	57.9 ± 3.0	54.7 ± 3.1	3.260	0.04

Δ *=* difference between the two evaluations; SD = standard deviation.

**Table 5:** Values found for the interobserver evaluation

	Evaluator 1 mean ± SD	Evaluator 2 mean ± SD	Δ	P
Strength	44.4 ± 3.8	44.8 ± 3.7	-0.460	0.82
Hand function	69.2 ± 5.2	68.6 ± 5.3	0.631	0.58
Mobility	77.0 ± 3.3	77.1 ± 4.0	-0.083	0.97
Activities of daily living	81.4 ± 2.8	82.1 ± 2.8	-0.651	0.55
Memory	75.7 ± 2.9	78.1 ± 2.9	-2.385	0.26
Communication	81.6 ± 3.4	85.0 ± 2.6	-3.398	0.14
Mood	52.8 ± 2.3	54.3 ± 1.7	-1.464	0.44
Social participation	57.9 ± 3.0	57.0 ± 2.9	0.972	0.49

Δ = difference between the two evaluations; SD = standard deviation.

The eight domains showed excellent internal consistency, with Cronbach’s alpha ranging from 0.75 to 0.99 (Table 6).

**Table 6:** Internal consistency of the eight domains

Domains	Cronbach’s alpha
Strength	0.86
Hand function	0.99
Mobility	0.92
Activities of daily living	0.93
Memory	0.89
Communication	0.88
Mood	0.75
Social participation	0.93

## DISCUSSION

The absence of instruments for assessing quality of life that have been translated and validated for use in Brazilian Portuguese among stroke patients has limited research in this field in this country. We decided to translate and culturally adapt SIS 2.0 and assess its reproducibility because this is an instrument that specifically assesses the impact of stroke on these patients’ quality of life. The protocol followed enabled proper translation of the original questionnaire, thus making it possible to use it for evaluating Brazilian patients with a diagnosis of stroke.

For an instrument analyzing the conditions of a patient to be considered appropriate for use within the scientific community, it needs to be reproducible.^15^ Reproducibility means that the same results are obtained when the questionnaire is applied at different times, to patients who present the same conditions. The SIS 2.0 questionnaire was reviewed regarding its intraobserver and interobserver agreement and calculation of intraclass correlations showed good agreement, both for application by the same investigator and for application by two different researchers.

For the intraclass correlation coefficient to demonstrate that an instrument is reproducible, the minimum acceptable value for this coefficient needs to be greater than or equal to 0.70 if the questionnaire is new, or greater than 0.80 if the questionnaire is old.^16^ In our study, the coefficients found were greater than or equal to 0.73, thus demonstrate the excellent reproducibility of the translated version of SIS.

Although there was no statistically significant intraobserver difference between the averages for some areas of the questionnaire in assessing the reproducibility, the important point is that the ICC was considered strong in all areas (0.75-0.99). Some of the responses to the questionnaire showed changes 15 days after the first application. This was probably because the questions that generated these responses related to feelings that could change over a 15-day period, for example, “I feel that I am a burden to others”. Another important point to be taken into consideration is that the possible answers often do not differ much between each other, for example “very” and “extremely”. Something may have been qualified as “very” in the first interview and, 15 days later, it may become “extremely.” Clinically, this change does not have any significant importance, but if taken in isolation, these two responses might be counted as if a change had occurred.

After the questionnaire had been applied during the interviews with the 10 patients in the adjustment phase, the lead researcher analyzed the main difficulties. This wide-ranging translation and adaptation process involving several steps made it possible to achieve linguistic equivalence between the words in the source language and target language. It was fully expected that certain problems would have to be addressed, such as the word *stroke*. From a semantic point of view, the word *stroke* (which literally means a “hit”) is a good example of how a difference between the source and target languages can distort meaning from a transcultural perspective. The word *stroke* was translated in the first version as “acidente vascular”, but all the patients suggested that this should be changed to the word “derrame”.

Regarding the assessment of reproducibility, the two researchers involved in the questionnaire had had specific training, thus making application of the questionnaires a homogeneous process. For it to be possible for questionnaires to be self-administered, they need to be simple and straightforward and must not lead to doubt among the patients.^17^ If the questionnaire fulfills these requirements, the need for training for the people who will apply it will be low, but this may lead to some possibility of creating a confounding factor.^17^

Regarding demographic characteristics relating to sex, age and schooling, the patients evaluated in our study were similar to those assessed in the original study that led to the SIS 2.0 questionnaire.^11^

Although patients who did not achieve the minimum score in the MMSE were excluded, this questionnaire enables the possibility that the interviewer can read it aloud, which is critical for its application to Brazilian populations, in which 40% are functional illiterates.^18^

The translation and cultural adaptation of SIS 2.0 contributes a further instrument that can be used in future studies on stroke patients, with the particular aim of assessing their quality of life. In general, this is a parameter that has been little reported in neurovascular assessments. Until now, most studies have used the SF-36 scale (Medical Outcomes Study 36-Item Short-Form Health Survey)^19^ to assess general wellbeing and the Barthel index^9^ to evaluate functional capacity, but these scales do not have the capacity to assess the quality of life of stroke patients.

## CONCLUSION

The resultant translation and adaptation of the SIS questionnaire (version 2.0) for use in the Portuguese language under Brazilian culture conditions was found to be easy for the patients to understand and had good reproducibility. This opens the possibility for its use among Brazilian stroke patients and allows evaluation of specific treatments.
